# Viral Diversity of Microbats within the South West Botanical Province of Western Australia

**DOI:** 10.3390/v11121157

**Published:** 2019-12-13

**Authors:** Diana Prada, Victoria Boyd, Michelle L. Baker, Mark O’Dea, Bethany Jackson

**Affiliations:** 1 School of Veterinary Medicine, Murdoch University, Perth, WA 6150, Australia; m.odea@murdoch.edu.au (M.O.); b.jackson@murdoch.edu.au (B.J.); 2 Health and Biosecurity Business Unit, Australian Animal Health Laboratories, CSIRO, Geelong, VIC 3220, Australia; vicky.boyd@csiro.au (V.B.); michelle.baker@csiro.au (M.L.B.)

**Keywords:** insectivorous bats, viral diversity, Western Australia, adenovirus, coronavirus, paramyxovirus, serology, next generation sequencing

## Abstract

Bats are known reservoirs of a wide variety of viruses that rarely result in overt clinical disease in the bat host. However, anthropogenic influences on the landscape and climate can change species assemblages and interactions, as well as undermine host-resilience. The cumulative result is a disturbance of bat–pathogen dynamics, which facilitate spillover events to sympatric species, and may threaten bat communities already facing synergistic stressors through ecological change. Therefore, characterisation of viral pathogens in bat communities provides important basal information to monitor and predict the emergence of diseases relevant to conservation and public health. This study used targeted molecular techniques, serological assays and next generation sequencing to characterise adenoviruses, coronaviruses and paramyxoviruses from 11 species of insectivorous bats within the South West Botanical Province of Western Australia. Phylogenetic analysis indicated complex ecological interactions including virus–host associations, cross-species infections, and multiple viral strains circulating concurrently within selected bat populations. Additionally, we describe the entire coding sequences for five alphacoronaviruses (representing four putative new species), and one novel adenovirus. Results indicate that viral burden (both prevalence and richness) is not homogeneous among species, with *Chalinolobus gouldii* identified as a key epidemiological element within the studied communities.

## 1. Introduction

In the past three decades, viral surveillance in wild bat populations has accelerated due to human fatalities and the socio-economic impacts of emerging infectious diseases with bat origins, including respiratory syndrome coronaviruses, and paramyxoviruses such as Nipah and Hendra viruses [1,2]. Describing viral diversity within bat communities facilitates early detection of direct and indirect zoonotic disease risks [3], which are intensified by the anthropogenic modification of the landscape, climate change and increased human–domestic animal–wildlife interactions [4,5,6]. In bats, this link has been clearly illustrated by the loss of suitable habitat for flying foxes, and the risks of spillover events either through increased viral shedding [7], or movements of bat communities into bushland–urban interfaces [8]. 

Viral surveillance of wild bat populations is also necessary for proactive conservation management of this diverse taxonomic group. Viral shedding can act as an indicator of broader environmental change driving host population stress [9,10], which can influence disease dynamics through behavioural and physiological changes in the host [10]. Recent findings suggest that higher loads of coronavirus may hinder the survival of bats infected with *Pseudogymnoascus destructans,* the causative agent of White Nose Syndrome which has had a devastating impact on the conservation of a range of North American microbat species [11]. Further, a relationship between reduced nectar-based resources in winter, and Hendra virus spillover has been described [12], arguably mediated by impacts on the host energy budget and therefore immune system, as well as dispersed foraging patterns of the host species. Given the plausible environmental and biological drivers of viral shedding in bats, characterising their viral diversity is a crucial first step to develop strategies to detect changes in pathogen dynamics with implications for public health and conservation [13]. 

Among the variety of viral groups harboured by microbats [14], *Adenoviridae*, *Coronaviridae*, and *Paramyxoviridae* are diverse viral families infecting a wide range of vertebrates, and include species of known zoonotic potential, with key examples being the precursors to the Severe Acute Respiratory Syndrome and the Middle East Respiratory Syndrome coronaviruses which originated in insectivorous bats [15,16,17]. Even though adenoviruses of bat origin have not been associated with human disease outbreaks, in-vitro experiments have demonstrated their capacity to infect other vertebrate cell lines, including human cell lines [18]. Additionally, cross species infections of adenoviruses from New World monkeys to humans and consequent human to human transmission have also been reported [19]. Even though microbats are not known to harbour paramyxoviruses of zoonotic or conservation concern, this viral group is of interest as Hendra and Nipah viruses, two paramyxoviruses of flying fox origin, are able to infect humans with fatal disease progression [20,21].

To date, only a few viral groups have been detected and characterised in Australian bats, with surveillance predominantly focusing on the role of Australian flying foxes as reservoirs of Australian bat lyssavirus (ABLV) and Hendra virus [22,23,24]. ABLV has been isolated from a single Australian microbat species [25], with evidence of previous exposure in several other microbat and megabat species [26,27]. Coronavirus surveillance in northern Australia resulted in the detection of novel Australian alpha- and betacoronavirus strains [28], and transmission dynamics for coronaviruses have been modelled for the microbat, *Myotis macropus* [29]. Viral characterisation of populations of *Miniopterus orianae bassanii* and *oceanensis* showed a high prevalence of herpesviruses but neither coronaviruses, filoviruses, henipaviruses, nor lyssaviruses were detected [30]. Currently, the pathogen–host associations, and potential human risks or bat conservation implications of viral diversity within Australian insectivorous bats remain relatively unknown. 

Here we describe the viral diversity of microbat communities within the South West Botanical Province (SWBP) of Western Australia, which is home to 13 species of microbats from two families, Vespertilionidae and Molossidae, including the locally endemic *Falsistrellus mackenziei*. We screened faecal samples for the presence of adenoviruses, coronaviruses and paramyxoviruses using targeted molecular approaches, and employed next generation sequencing (NGS) and serological protocols to enable a broader characterisation of viral diversity. 

## 2. Materials and Methods 

### 2.1. Study Area

**Figure 1:**
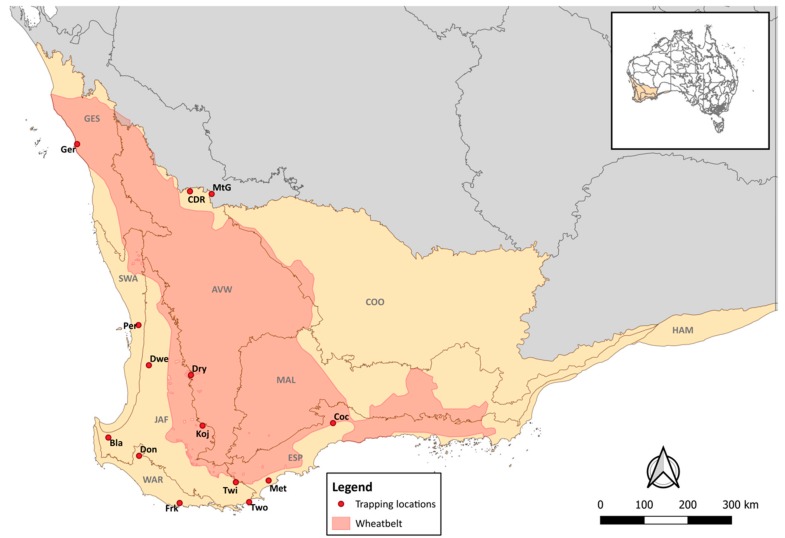
The South West Botanical province (SWBP), highlighted in yellow, sampling sites and extent of the Australian wheatbelt are displayed. The SWBP encompasses nine bioregions, Avon Wheatbelt (AVW), Coolgardie (COO), Esperance Plains (ESP), Geraldton Sandplains (GES), Hampton (HAM), Jarrah Forest (JAF), Mallee (MAL), Swan Coastal Plain (SWA), and Warren (WAR).

The South West Botanical Province (SWBP), a global biodiversity hotspot [31], contains nine bioregions covering approximately 44 million hectares [32] and has a predominantly Mediterranean-type climate. Landscapes in the area are floristically diverse and have significantly changed due to anthropogenic activities [32]. Remnant natural cover on the east and west of the region is separated by the extensive monoculture known as the Western Australian wheatbelt (Figure 1). 

Sampling took place to the south-west and the north-east of the province, with the 14 sampling sites falling on the east and west boundaries of the wheatbelt. The south-western sites were distributed across four bioregions: Swan Coastal Plain, Jarrah Forest, Warren and the Esperance Plains. The north-eastern sites were predominantly within the Avon bioregion (Figure 1). 

### 2.2. Sample Collection

All sampling was approved by the Department of Biodiversity Conservation and Attractions, permits 08-001359-1, and CE005517. Capture, handling and sampling procedures were approved by the Murdoch University Animal Ethics Committee (R2882/16). Sampling occurred over two summers between November and April (2016–2018) using a cross-sectional sample design. The sites within the Avon bioregion were predominately sampled during the first year and the southwest sites during the second.

Bats were captured using harp traps and mist nets set up near water bodies or across forest tracks. Captured individuals were placed inside clean calico bags for at least ten minutes to allow time for defecation. Each individual was weighed and identified to species or genus level, using morphological features [33]. 

Collected faecal pellets were placed into 250 µL of RNAlater^®^ (Ambion, Life Technologies, Carlsbad, CA, USA) and kept at either room temperature or 4 °C for a maximum of three days. Upon arrival to the laboratory, samples were stored at −80 °C until processing. A total of 10 µL of blood was collected, which was diluted 1:10 in phosphate buffered saline (PBS) and kept at 4 °C before processing

### 2.3. Molecular Analysis

Faecal samples were homogenised in the preservation solution by vigorous vortexing and centrifuged at 17,000 × g. A total of 50 µL of the supernatant was used as starting material for all extractions, using a Magmax viral RNA extraction kit (Ambion, Life Technologies, Vilnius, Lithuania) according to the manufacturer’s instructions. 

Screening of coronaviruses and paramyxoviruses consisted of a nested and semi-nested reverse transcription PCR (RT-PCR) amplifying a segment of the RNA-dependent RNA polymerase (RdRp). 

Coronavirus screening was performed with two primer sets that amplify different sections of the RdRp gene. These assays were run in parallel in order to increase sensitivity [34]. Optimisation of the paramyxovirus assay involved testing two previously published primer sets [35] on a sample subset (*n*= 48). The first set (pan-paramyxovirus) targeted all genera within the family and the second was specific to *Morbillivirus, Respirovirus,* and *Henipavirus* (Mor-Res-Hen primer set). Following an assessment of the results, subsequent screening was carried out using the group-specific primer set (Mor-Res-Hen primer set). Screening of adenoviruses involved the amplification of a section of the DNA polymerase gene (DNApol) (Table 1).

Reverse transcription PCR amplifications were performed using SuperScript III One-Step with Platinum Taq (Invitrogen, Thermofisher, Carlsbad, CA, USA). Additional PCRs were carried out with MyTaq PCR mix (Bioline, London, UK). Annealing temperatures are reported in Table 1. 

Amplicons were visualised in 2% agarose gels stained with SYBR™ safe (Invitrogen, Thermofisher, Carlsbad, CA, USA). Products of the expected size were purified using Agencourt AMpure XP beads (Beckman Coulter, Brea, CA, USA). All fragments underwent Sanger sequencing in forward and reverse directions with the BigDye Terminator v3.1 Cycle Sequencing Kit (Applied Biosystems, Carlsbad, CA, USA) on an ABI PRISM 3130 DNA Analyser (Applied Biosystems, Carlsbad, CA, USA). 

For full-length reconstruction of viral genomes, faecal nucleic acid samples were subject to sequence independent single primer amplification (SISPA) based PCR to capture RNA and DNA viral material [36]. Library preparation was performed using a Nextera XT library kit (Illumina, San Diego, CA, USA) as per the manufacturer’s instructions, and sequencing was performed on a NextSeq 500 using a 2x150 mid-output flowcell (Illumina, San Diego, CA, USA). De novo assembly of genomes was performed using SPAdes [37] with the *–careful* parameter enabled. Contigs were searched against the GenBank non-redundant protein database using DIAMOND v0.9 [38], and those returning significant similarity to viral sequences (E value ≤10^-5^) were retained. Contigs matching coronavirus and adenovirus sequences were extracted and examined manually by ORF prediction followed by BLASTn and BLASTp search. For contigs in which entire coding sequences were present, manual genome annotation was performed using Geneious v10.2.6 (BioMatters, Auckland, New Zealand). 

### 2.4. Phylogenetic Analysis

Sequence read data were manually checked and trimmed in Geneious v10.2.6 (Biomatters, Auckland, New Zealand), and subjected to BLASTn search to verify the identity of the sequenced amplicons. Reference sequences, as recognised by the International Committee on Taxonomy of Viruses (ICTV) and other publicly available sequences, were retrieved from GenBank and aligned to the sequences generated by this study using MUSCLE [42] for the *Adenoviridae* and *Coronaviridae* alignments and MAFFT [43] for *Paramyxoviridae*.

Nucleotide-based phylogenetic relationships were inferred from maximum likelihood and bayesian analysis, based on the general time-reversible evolution model with gamma-distributed rate variation among sites and a proportion of invariable sites. Maximum likelihood trees were constructed in Mega X [44]; all gaps and missing data were subject to total deletion and trees bootstrapped 1000 times. Bayesian trees were constructed in MrBayes v.3.2 [45]. The default settings were employed to set the prior probabilities on the model parameters. Two runs were performed for 1,000,000 generations and convergence of the posterior probabilities were assessed by checking the standard deviation of split frequencies and the resulting PSRF statistics. Additional generations were added until the standard deviation reached a value between 0.01 and 0.03. 

Phylogenetic analyses for five whole coronavirus genomes constructed from NGS data were performed on the polyprotein 1ab and spike gene amino acid sequences. Representative coronavirus sequences were retrieved from GenBank and aligned using MUSCLE [42]. Phylogenetic relationships were inferred by constructing maximum likelihood trees using the PROTGAMMAWAG model with 1000 bootstraps in RAxML [46]. 

### 2.5. Serology

Samples were tested for reactivity to four paramyxovirus and two betacoronavirus antigens in an indirect-binding Luminex^®^ assay at a final working dilution of 1:50. All serological testing was performed at the CSIRO Australian Animal Health Laboratory (AAHL; Victoria, Australia). The assay coupled soluble G (sG) glycoproteins from Hendra virus (HeV), Nipah virus (NiV) and Cedar virus (CedV), and nucleoproteins (N) from Tioman virus (TioV), SARS, and MERS coronaviruses to individual microsphere sets as previously described [47]. 

A total of 662 samples were obtained for serological analysis. Those collected during the first season were pooled one in three (*n*= 246) or one in four (*n*= 24). Positive pools were then tested at the individual sample level where possible. Pooling has shown not to have a diluting or detrimental effect on the assay result [48]. All samples collected during the second season (*n*= 392) were tested individually. Median Fluorescence Intensity (MFI) was read using a Bio-Plex 200 instrument (Bio-Rad laboratories Hercules, CA, USA). Positive and non-template controls were included in all runs. Positive sera were of non-bat origin from naturally or experimentally infected animals. Serology results from this study are referred to as paramyxovirus-like (PaV), and betacoronavirus-like (βCoV).

As the Luminex assay has not been validated for microbat species due to the lack of species-specific positive controls, the MFI threshold to differentiate positive and negative samples was set up at 1000 MFI, which is at least three times the mean MFI of negative sera. It is based on previous studies published by the AAHL and elsewhere using the same Bio-Plex platform on sera from other bat species with values below 250 MFI considered negative [49,50,51,52]. 

### 2.6. Prevalence Estimates 

The Wilson’s method [53] was used to estimate prevalence values and 95% confidence intervals in R package *epitools* [54]. Estimates for the molecular data were stratified by viral family, and host species. Seroprevalence was calculated using all samples within the serology dataset (*n*= 662). Positive results in pooled samples were assigned to a single individual within each pool. 

Species-specific prevalence estimates were derived from samples tested individually and calculated for sample sizes greater than 20 individuals as estimates with a small denominator provide meaningless values. Differences between prevalence values were assessed using the Fisher Exact test as implemented in R.

## 3. Results

A total of 571 faecal samples were collected from 11 species of microbats, with *Chalinolobus gouldii* (*n*= 232) and *Vespadelus regulus* (*n*= 141) having the largest representation in the dataset. Details on number of faecal and blood samples per species and collection sites are shown in Table 2.

### 3.1. Serology

Resulting overall antibody prevalence estimates were generally low for all serology assays. The highest values were observed for SARS-CoV at 5.8% (95% CI 4.3–7.9%) and CedV at 5.3% (95% CI 3.8–7.3%), and the lowest for NiV at 1.3% (95% CI 0.7–2.6%) and MERS-CoV at 1.5% (95% CI 0.8–2.7%). Sample sizes, overall seroprevalance estimates and associated confidence intervals are shown in Table 3.

PaV-like and βCoV-like antibody responses were detected in eight and six species, respectively, (Table 4) but only *C. gouldii* and *V. regulus* reacted to all antigens. Species-specific antibody prevalence values were also low, with the highest value recorded for CeV antibodies in *C. morio* at 10.3% (95% CI 4.8–20.7%) and βCoV antibodies in *N. gouldi* at 10% (95% CI 5.1–20%). 

### 3.2. Faecal PCR Analysis

Overall, the highest prevalence of viral nucleic acid shedding was observed for coronaviruses at 18% (95% CI 15–22%), followed by adenoviruses at 11% (95% CI 8–14%) and paramyxoviruses at 3% (95% CI 2–5%). All viral families were predominantly amplified from four species, *C. gouldii*, *C. morio, N. geoffroyi*, and *V. regulus*, with the former two species showing the highest prevalence estimates for adenovirus DNA and coronavirus RNA (CoV-RNA) (Table 5). 

Prevalence values across species were significantly different for adenoviruses and coronaviruses but not for paramyxoviruses. This differentiation was driven by the high prevalence in *Chalinolobus spp*, compared to all other species. 

### 3.3. Summary of Virus Prevalence in Relation to Serology

Seroprevalence values for βCoV antibodies markedly differed from CoV-RNA prevalence estimates, especially for *C. gouldii*, *V. regulus* and *C. morio*. These three species showed the greatest levels of CoV-RNA shedding with values ranging from 12% to 36% (Table 5), while the corresponding βCoV seoprevalence was only between 1.6% and 6.7% (Table 4). To the contrary, PaV-RNA prevalence rates for these three species were comparable to their observed seroprevalence ranges. Thus, *C. morio*, which showed the highest PaV-RNA prevalence at 9% (95% CI 3–21%), had a seroprevalence range between 3.3% and 10.3%. *C. gouldii* showed a PaV-RNA shedding prevalence of 4% (95% CI 2–7%) and a PaV-seroprevalence ranging between 3% to 6%, and in *V. regulus* PaV antibody prevalence varied between 0.6% to 6.1% and the corresponding PaV-RNA shedding was 1.4% (95% CI 0.4–5%). Individuals that were seropositive in either coronavirus or paramyxovirus assays yielded a negative PCR result for either family, except for a single *C. morio* which was RNA and antibody positive for coronaviruses. 

The detection rate of co-infections was low and limited to *Chalinolobus spp*. CoV and PaV- RNA were found in three individuals, CoV-RNA and AdV-DNA were detected in 17 individuals, and all three viral families were amplified from a single sample. 

### 3.4. Phylogenetic Analysis and Whole Genome Sequencing

Phylogenetic analysis showed a diverse number of coronavirus, adenovirus and paramyxovirus strains circulating within microbat populations of the SWBP. Two different strains of coronavirus were detected in *C. gouldii*, *V. regulus* and *N. geoffroyi*. For adenoviruses, three strains were observed in *C. gouldii* and two in *N. geoffroyi*, and two paramyxovirus strains were amplified in *C. gouldii* and *V. regulus*. GeneBank accession numbers of representative sequences generated by this study are reported in Table S1. Resulting tree topologies were similar under the maximum likelihood and Bayesian analyses. Additionally, a total of six full genomes were described: five coronaviruses and one adenovirus. Details are provided in the sections below. 

In general, a host species–strain association independent of the sampling region was observed for all viral families. In the majority of cases, individuals captured between 50 to 600 km apart, shared the same viral strain. However, for *C. morio*, a geographic clustering pattern was observed in the coronavirus analysis, with cluster delineation appearing to be based on the south-west and north-east sampling sites. 

#### 3.4.1. Adenovirus

**Figure 2:**
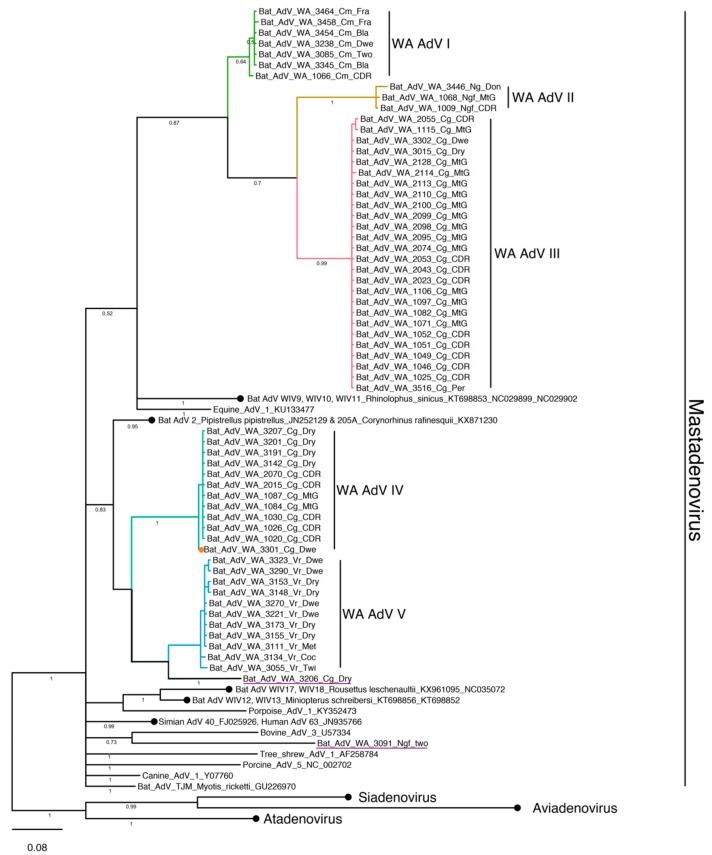
Bayesian phylogenetic analysis of adenovirus sequences based on a fragment of the DNApol gene. Tree was constructed with Mr Bayes, posterior probabilities of mayor partitions are reported below branches. Clades identified in this study are coloured, naming convention represents the unique ID for each individual followed by the first letter of the genus, the first letter of the species and the geographical trapping site. A full adenovirus genome was amplified from the sample marked by an orange circle and samples that did not cluster within any of the identified WA clades are underlined. GeneBank accession numbers for representative sequences produced by this study are reported in Supplementary Table S1. Within *Mastoadenovirus*, clades of reference samples were collapsed for brevity, black circles, and accession numbers are shown following strain names. Genbank accession numbers for representatives of *Siadavirus*, *Aviadvirus* and *Atadenovirus* are reported in Supplementary Table S2.

The final working alignment for adenovirus amplicons contained 61 sequences of 228 bp. All amplified strains grouped into five clades (I-V) within the genus *Mastadenovirus* (Figure 2).

Most clades were dominated by strains detected from individuals of the same species sampled at different locations. WA AdV clade I consisted of seven *C. morio*, clade III and IV of 26 and 12 *C. gouldii* respectively, and clade V of 11 *V. regulus*. Clade WA AdV II contained three highly similar sequences, amplified from two different species, *N. geoffroyi*, and *N. gouldi*.

Additionally, two separate sequences which did not cluster directly within the WA AdV clades, were detected from single *C. gouldii* and *N. geoffroyi* individuals. The former was most similar to those within WA AdV V and the latter, grouped with Bovine AdV 3 (U57334). 

WA AdV clades IV, V and sequence 3206-Cg-Dry clustered with sequences amplified from other Vespertilionidae species, Bat AdV 2 (JN252129) and Bat AdV 205A (KX871230), a pattern not observed for the remaining sequences. Under the Bayesian phylogenetic analyses, the remaining clades grouped with sequences amplified from *Rhinolophus sinicus* (KT698853, NC029899, NC09902) a member of Rhinolophidae, while under the maximum likelihood approach there was not a clear association to any other sequence of bat origin. 

A single full-length adenovirus genome was obtained from a *C. gouldii* (Bat AdV WA 3301 Cg Dwe, clade IV, GenBank accession MK472072). The genome was 37,617 nucleotides in length, with inverted terminal repeats of 288 bp and 293 bp at the 5’ and 3’ termini, respectively. A BLASTn search on the full-length genome returned most similarity to Human adenovirus D isolate (KF268332.1) with 72% identity over a 32% query cover. Of the 27 putative open reading frames identified, the conserved DNA polymerase protein showed the greatest identity (62%) to canine (AP000050.1), equine (ANG08548.1) and bat (YP_004782100.1) adenoviruses. The hexon protein appeared to be more evolutionarily conserved with the highest similarity to Bat mastadenovirus G (YP009325345.1) (query coverage 100%, identity 75%), identified from *Corynorhinus rafinesquii*, a member of Vespertilionidae.

#### 3.4.2. Coronavirus

Coronavirus RNA was detected in 102 samples. Significant differences were observed in sensitivity between the two primer sets used for the screening. The CoV primers [40] detected 98 positives while the PQL primers [41] detected only nine. Since these two primer sets amplify different regions of the RdRp gene, only those sequences successfully amplified with the CoV primers were used to build the alignment, which consisted of 91 sequences of 396 bp. 

**Figure 3:**
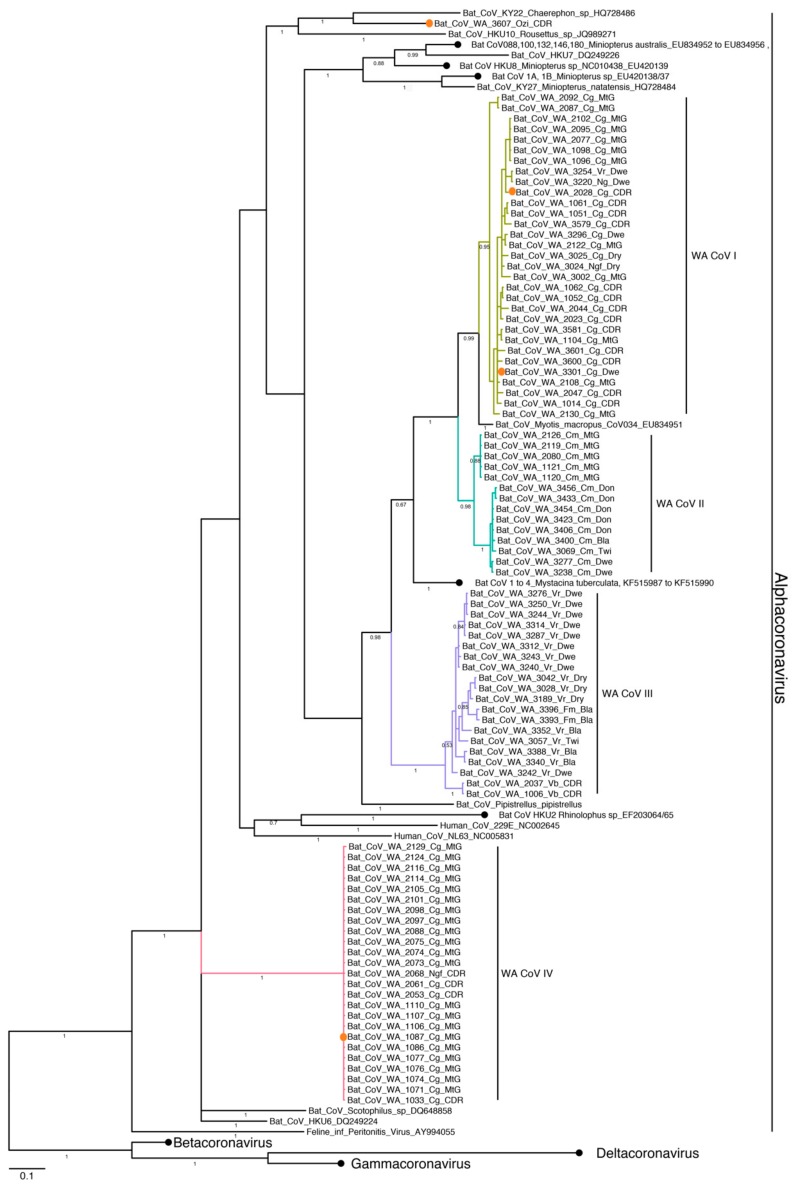
Bayesian phylogenetic analysis of coronavirus sequences based on a fragment of the RdRp gene. Tree was constructed with Mr Bayes, posterior probabilities of major partitions are reported below branches. Clades identified in this study are coloured, naming convention is based on the unique ID for each individual followed by the first letter of the genus, the first letter of the species and the geographical trapping site. Full coronavirus genomes were amplified from the samples marked by an orange circle. GenBank accession numbers for representative sequences produced by this study are reported in Supplementary Table S1. Within *Alphacoronavirus*, clades of reference samples were collapsed for brevity, black circles, and accession numbers are shown following strain names. GenBank accession numbers for representatives of *Betacoronavirus*, *Deltacoronavirus* and *Gammacoronavirus* are reported in Supplementary Table S2.

Sequences grouped into four clades (I-IV) within the genus *Alphacoronavirus* (Figure 3). There was a clear host species dominance within each clade, WA CoV I and IV consisted predominantly of *C. gouldii* (*n*= 28, 24), WA CoV II of *C. morio* (*n*= 14), and WA CoV III of *V. regulus* (*n*= 16). Additionally, a single sequence was amplified from *Ozimops sp*. Despite the observed species-specific dominance, clades also included highly similar sequences detected from a minority of other species. Consequently, strains from two *Nyctophilus spp*, and one from *V. regulus* clustered with the 28 *C. gouldii* sequences within WA CoV I, two *F. mackenziei*, and two *V. barvestocki* clustered within the *V. regulus* clade (WA CoV III), and a sequence amplified from *N. geoffroyi* was identical to *C. gouldii* sequences within WA CoV IV.

Phylogenetic analysis also showed that all CoV groups were similar to sequences amplified from members of the same host family. Accordingly, the *Ozimops sp* sequence clustered with a Molossidae sequence (*Chaerephon sp*. HQ728486), and all the remaining clusters were most similar to sequences from members of Vespertilionidae. WA CoV clades I, II and III grouped with previously described strains from Australian and New Zealand vespertilionid bats (EU834951, KF545987-90).

Five full alphacoronavirus genomes were amplified (GenBank accession numbers MK472067–MK472071). Three genomes were obtained from *C. gouldii* individuals, 2028-Cg-CDR and 3301-Cg-Dwe, representing clade CoV I and Bat CoV WA 1087-Cg-MtG, within clade CoV IV. Additional genomes were amplified from a pooled faecal sample of *V. regulus* (Bat CoV WA Vrpool-Dwe) and one from *Ozimops sp* (Bat CoV WA 3607-Ozi-CDR). Genome sizes ranged from 27,405 nt to 28,171 nt in length, with all genomes containing the ORF1ab translated as a result of the presence of the ribosomal frameshift signal UUUAAAC. In addition to ORF1a, ORF1b and ORF1ab, all genomes contained coding regions for the spike protein, envelope protein, membrane glycoprotein, nucleocapsid and ORF3. 

**Figure 4:**
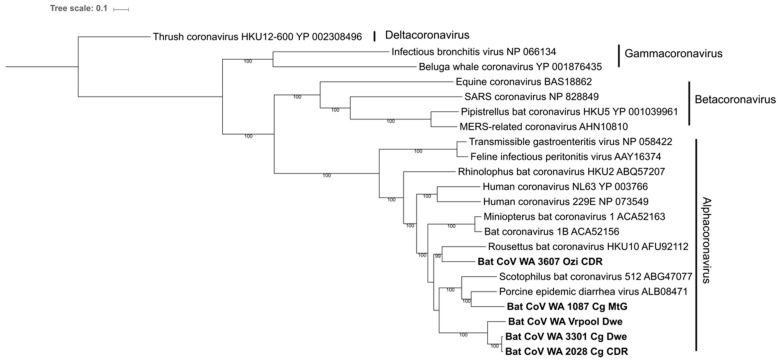
Maximum likelihood phylogenetic analysis of the pp1ab amino acid sequences derived from five coronavirus genomes. The tree was constructed in RAxML using the PROTGAMMAWAG model with 1000 bootstraps; supports above 80% are shown below each branch. Naming convention for the sequences generated in this study is based on the unique ID for each individual followed by the first letter of the genus, the first letter of the species and the geographical trapping site.

Phylogenetic analysis of the pp1ab amino acid sequences (Figure 4) and spike gene sequences (Figure S1) showed that genomes from clade CoV I clustered with the genome amplified from the pooled *V. regulus* faecal samples, while the *Ozimops sp* genome clustered with *Rousettus* bat coronavirus HKU10 (AFU92112). Even though these viruses predominantly clustered with other bat viruses, the genome representing clade CoV IV clustered with Porcine Epidemic Diarrhoea virus (PEDV) (ALB08471), with an amino acid identity of 74.1% for the pp1ab region and 67.7% for the spike protein.

Analysis of concatenated amino acid domains from ORF1ab (NSP3, NSP5, NSP12, NSP13, NSP14, NSP15, and NSP16) showed that the two samples from clade CoV I had over 99% identity to each other while levels of identity among the remaining three genomes ranged from 67.8% to 85.5%. Levels of identity to other coronaviruses also resulted in identity levels below 90%. Therefore, the five genomes reported here represent four putative new coronavirus species, as per the International Committee on the Taxonomy of Viruses guidelines.

#### 3.4.3. Paramyxovirus

**Figure 5:**
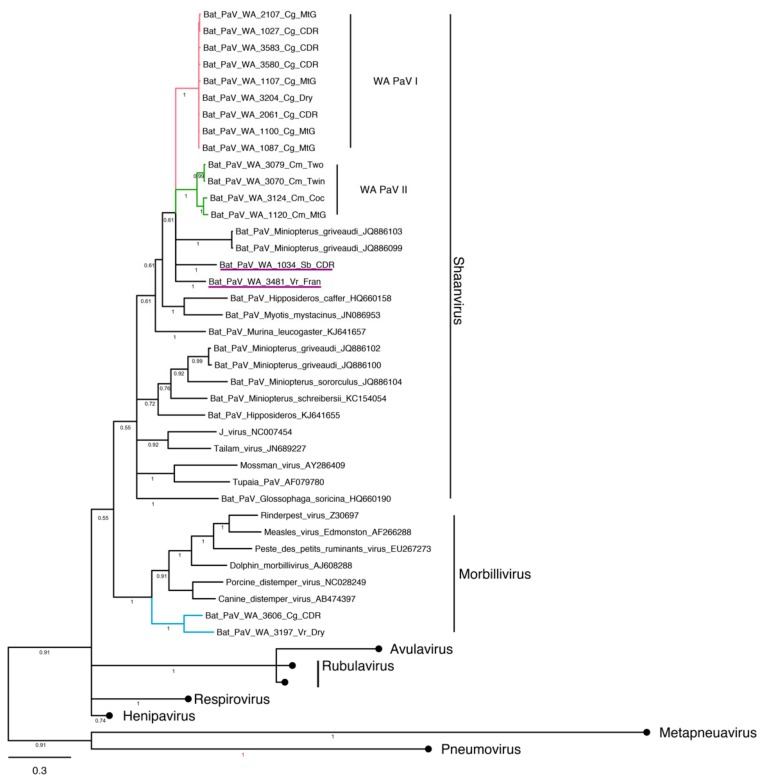
Bayesian phylogenetic analysis of paramyxovirus sequences based on a fragment of the RdRp gene. Tree was constructed with Mr Bayes, posterior probabilities of major partitions are reported below branches. Clades identified in this study are coloured, naming convention for sequences produced by this study is based on the unique ID for each individual, followed by the first letters of the genus, the species and the geographical trapping site. Samples that did not cluster within any the identified WA clades are underlined. GeneBank accession numbers for representative sequences produced by this study are reported in Supplementary Table S1. Clades of reference samples were collapsed for brevity, black circles, GenBank accession numbers for representatives of *Rubulavirus*, *Avulavirus*, *Respirovirus*, *Henipavirus*, *Metapneuavirus* and *Pneumovirus* are reported in Supplementary Table S2.

Paramyxovirus RNA was detected in 18 samples. The working alignment was 443 bp long and contained 17 sequences. Sequences clustered by host species, forming two main clades (Figure 5). WA PaV I consisted of nine strains amplified from *C. gouldii*, and WA PaV II consisted of four sequences from *C. morio*. These two clades and two additional sequences amplified from a single *S. balstoni* and a *V. regulus* clustered within the recently proposed genus Shaanvirus [55] and were phylogenetically related to other reported sequences amplified from host species of the same family. Two distinct sequences detected in *V. regulus* and *C. gouldii* clustered within the genus *Morbillivirus*. To further investigate the relationships to other bat morbilliviruses, these two samples were amplified with the Pan-paramyxovirus primer set. Only one sample was successfully sequenced, and the resulting sequence clustered within Shaanvirus instead of *Morbillivirus*. 

No contigs with homology to known paramyxovirus species were found following next-generation sequencing analysis, inclusive of two samples paramyxovirus positive on RT-PCR (3197- Vr-Dry and 1087-Cg-MtG).

## 4. Discussion

Here we present a comprehensive characterisation of the viral diversity within Australian insectivorous bat communities, providing the first records of adenovirus and paramyxovirus shedding in Western Australian bats, as well as building on the current knowledge of coronaviruses in Australian microbats. Our results indicate that disease burden varies within bat species assemblages, and *C. gouldii* appears to be a key epidemiological element within the studied communities, sustaining the greatest viral richness and shedding. This study shows that WA microbat populations host a variety of viral families and sustain multiple viral strains concurrently, in line with other studies [56,57,58,59,60,61]. Our results are in contrast to the only additional Australian initiative to date aiming at characterizing viral diversity in microbats, where only herpesviruses were detected [30]. Difference in the discovery rate between these two studies may be due to the number of species surveyed (two subspecies vs. 11 species) as well as sample type (oral swabs vs. faeces), with oral swabs likely to carry lower levels of viral RNA/DNA, thus decreasing the sensitivity of molecular assays.

In general, phylogenetic analysis revealed an expected strain–host specificity independent of geographical distance, in line with previous findings for the targeted viral families [1,28,56,57,58,62,63,64,65,66,67,68]. At a finer scale, clustering patterns of coronavirus strains for *C. gouldii* and *C. morio,* may reflect the ecological connectivity between host populations. Host tropism over a wide spatial scale was particularly evident in the high similarity (99% identity) of the *C. gouldii* coronavirus genomes amplified from individuals captured at sites 350 km apart, while a partition between sites north and south of the wheatbelt was observed for coronavirus strains amplified from *C. morio*. Even though such partition was not reflected by either the adenovirus or paramyxovirus phylogenetic analysis, genetic or capture/recapture studies would clarify the current and historical connectivity of bat populations within the region, and their relationship to observed host–pathogen associations. 

Phylogenetic analysis of coronavirus sequences showed that species-specific groups harboured a minority of identical or similar strains which were amplified from sympatric individuals of different genera, providing evidence for the occurrence of historic cross-species transmission events. For example, within clade WA CoV IV, a sequence amplified from *N. geoffroyi* was identical to those amplified from *C. gouldii,* with all individuals captured at the same location, while sequences amplified from *F. mackenziei* clustered with the clade dominated by *V. regulus*. Previous cross-species infections of bats have mainly been observed in cave-dwelling species that share roosting sites, with viral transmission plausible within this context [28]. However, bat species in the current study predominantly roost in tree hollows and decorticating bark, although cave colonies of *C. morio* are also known [33]. Given the predominant faecal–oral or respiratory transmission of CoV, the observed strain clustering patterns suggest that *C. gouldii* may either share roosts concurrently or use them alternatively with *Nyctophilus* species and *V. regulus,* and a similar interaction may occur between *F. mackenziei* and *V. regulus*. This is supported by studies showing that despite forest bats having strong intra and interspecific variation in roost requirements [69,70], some species also exhibit considerable flexibility in roost selection [71]. Additionally, unpublished records from the bat box monitoring program at Organ Pipes National Park (Victoria, Australia) document the occasional co-roosting of *V. regulus* with *C. morio* or *C. gouldii* [72]. 

Interestingly, the phylogenetic analysis of the spike protein from coronavirus genome Bat CoV 1087-Cg-MtG showed a higher level of similarity to Porcine Endemic Diarrhoea virus (PEDV) than to any other virus of bat origin. This raises the possibility of a spillover risk, as the spike protein determines cell tropism. For instance, the emergence of the alphacoronavirus Swine Acute Diarrhoea Syndrome (SADS) in Chinese swine, is a recent example of a cross-taxa infection likely originating from an insectivorous bat coronavirus [73]. Both SADS and HKU2 bat coronavirus, the likely strain of origin, share 86% identity across the spike protein gene. However, the nucleotide identity of only 67.7% between the spike protein of Bat CoV 1087-Cg- MtG and PEDV suggests that direct infection to swine would be unlikely, and intermediate host jumps would likely be required. 

Cross-species infections were not generally supported by the paramyxovirus or adenovirus phylogenetic analysis. However, we detected an identical single adenovirus strain from *N. geffroyi* and *N. gouldi* collected at two separate regions. The small amplicon used for phylogenetic analysis may fail to discern a taxonomic separation between these two closely related species, although previous studies have reported the occurrence of divergent and cosmopolitan AdV strains [57,74,75]. However, evidence supporting this hypothesis has been based on a small amplicon and further studies on the co-evolution of adenoviruses and bats will benefit from whole genome analysis.

Host tropism was also supported by the full-length adenovirus amplified from *C. gouldii*. In general, the genome had low levels of identity to other available adenovirus genomes. A strong host–strain association was also supported by the significant difference of the fiber protein, which determines cellular tropism, to other sequences in GenBank, including those derived from bat adenoviruses. Additionally, identity analysis using the conserved hexon region was able to better identify this strain as of bat origin over DNApol or the fiber protein. This supports previous findings that tree topologies based on the hexon gene better reflect the phylogenetic relationships of the host than the DNApol gene [56,57,75]. 

Despite adenoviruses being detected in a smaller number of microbat species compared to coronaviruses (five versus eight species), they showed a similar level of strain richness, supporting the emergent consensus that this family encompasses a greater diversity than previously thought [56,68,76,77,78]. The relationship of the first three adenovirus clades to other published bat sequences was not resolved, likely due to the lack of comparable sequences. Despite the availability of several published *Adenoviridae* sequences, these represent alternative sections of the DNApol gene, highlighting the importance of unified protocols for the screening of wildlife populations to allow global comparisons [79]. 

Molecular detection of paramyxoviruses in faeces found a crude prevalence of 3% in line with previous reports [80,81]. We observed greater reactivity to antigens from Australian paramyxovirus species (Hendra virus and Cedar virus) than to those found in Asia (Nipah virus and Tioman virus). This pattern may be indicative of the closer relationship expected between Australian henipaviruses than to geographically distant viral species, and is consistent with cross-reactivity experiments on Hendra and Nipah antigens showing variability of the binding response despite the close relationship between these two viruses [47]. 

The serological results illustrate the challenges of sero-surveillance in novel species in the absence of a validated species-specific assay [82]. For example, RNA- and seroprevalence differed for the coronaviruses, in contrast to the paramyxoviruses where the RNA- and seroprevalence were consistent. A high prevalence of coronavirus RNA shedding suggests endemic infection of the studied communities, a finding not supported by the low seroprevalence. Such discrepancy is most likely caused by the lack of sensitivity of the serological assay at detecting alphacoronavirus antibodies, as it comprises SARS and MERS specific antigens. It could be argued that the use of alphacoronavirus antigens may have been more appropriate as a serological screening tool. However, given the number of betacornaviruses which are precursors of significant zoonotic viruses, we deemed it important to perform serological surveillance for this group of viruses. The serological assays employed here were developed for Australian flying foxes and have not been validated in insectivorous bats. Therefore, a true epidemiological picture cannot be resolved without further validation. Furthermore, unlike studies focusing on larger bats, the small amount of blood that may be ethically drawn from microbats makes it challenging to carry out confirmatory tests on seropositive samples. 

Viral shedding, antibody reactivity, and viral richness varied across species, suggesting that viral burden is not homogeneous within bat communities, but harboured by a minority of key species. In this study, *C. gouldii* and *C. morio* consistently showed the highest levels of viral shedding across all viral families despite the marked differences in total sample size (232 vs. 45 individuals). Differential viral burden within bat communities has also been hypothesised for Australian pteropid bats as reservoirs of Hendra virus, with longitudinal studies indicating that spillover events are more likely associated with two of the four species of Australian flying foxes, despite all being known reservoirs of the virus [83]. 

While multiple studies discuss the life-traits that make bats competent viral reservoirs compared to other taxa [84,85,86], only a few have specifically looked into the drivers of pathogen persistence and richness within bat communities [87,88,89]. Such studies investigated the relationship between viral richness and geographic range, colony size, population fragmentation, body size, and threat status. In general, the role of home range appears to be an important driver of viral richness [88,89], which is congruent with the global pattern that widely distributed species at high densities harbour a greater diversity of pathogens [90,91]. Equally, phylogenetic affinity has been suggested as an underlying factor of viral richness, as pathogens are more likely to host-switch with closely related species [86,92]. Within this context, *C. gouldii* could be proposed as an important viral reservoir within bat communities of the SWBP. This species showed the greatest viral richness with a total of seven strains detected, it has a wide distribution being commonly found in a variety of habitats including natural, rural and urban environments [33], and it appears to have a higher level of tolerance to urbanisation than other Australian microbat species [93,94]. 

High viral prevalence and richness were expected from *A. australis*, as this species has a wide distribution [33], it is capable of covering long distances, and its suspected migratory behaviour [95] would facilitate contact with species with more limited ranges, thus making it a potential viral reservoir and super-spreader at large landscape scales. However, the small sample size (*n*= 9) precluded any in-depth characterization of the viral diversity within this group and its role as a super-spreader at large scales warrants further research. 

Since temporal fluctuation of viral load has been documented in virus surveillance of north American and European microbats [96,97] as well as Hendra shedding in Australian peteropid bats [22,98], longitudinal studies would be required to fully understand the viral shedding and viral–host associations observed here. Furthermore, evidence of higher viral loads as indicators of individual and population scale stress [10] provides a good example of how long-term monitoring of viral prevalence can act as an indicator of environmental pressure on bat communities, particularly with regards to climate change where habitat contracture and distributional shifts may bring together populations harbouring novel viral strains. This approach would allow bat viral research to move beyond prediction of zoonotic disease emergence and be used as a management tool for monitoring the health of cryptic bat communities and the environment as a whole. 

## Figures and Tables

**Table 1:** Primer sets used in this study for the screening of Adenoviruses, Coronaviruses, and Paramyxoviruses.

Target Group	Target Region	Primer Name	T °C	Amplicon Size	Reference
Adenovirus	DNA polymerase gene (DNApol)	F1	55	205	[39]
		R1			
		F2	55		
		R2			
Coronavirus	RNA-dependent RNA polymerase gene (RdRp)	CoV-Fwd1	53	400	[40]
		CoV-Rvs2			
		CoV-Fwd2	50		
		PLQ-F1	50	300	[41]
		PLQ-R1			
		PLQ-F2	50		
		PLQ-R2			
Paramyxovirus	RNA-dependent RNA polymerase gene (RdRp)	PAR-F1	53	500	[35]
		PAR-R			
		PAR-F2	50		
		RES-MOR-HEN-F1	53	500	
		RES-MOR-HEN-R			
		RES-MOR-HEN-F2	50		

**Table 2:** Details of species sampled, number of faecal and serum samples collected per species and collection sites.

Family	Species	N Faecal	N Serum	Trapping Locations
Vespertilionidae	*Chalinolobus gouldii*	232	264	MtG, CDR, Coc, Dry, Dwe, Koj, Per
	*Chalinolobus morio*	45	66	MtG, Bla, CDR, Coc, Don, Dry, Dwe, Frk, Met, Twi, Two
	*Falsistrellus mackenziei*	11	7	Bla, Dwe
	*Nyctophilus geoffroyi*	51	50	MtG, Bla, CDR, Coc, Don, Dry, Dwe, Ger, Per, Two
	*Nyctophilus gouldi*	56	72	Bla, Don, Dry, Dwe, Per, Two
	*Nyctophilus major*	10	6	Bla, CDR, Dry, Dwe, Frk
	*Scotorepens balstoni*	9	8	MtG, CDR
	*Vespadelus baverstocki*	4	5	MtG, CDR
	*Vespadelus regulus*	141	170	Bla, Coc, Don, Dry, Dwe, Frk Met, Per, Twi, Two
Molossidae	*Austronomus australis*	9	11	CDR
	*Ozimops sp*	3	3	CDR

**Table 3:** Overall seroprevalence to six antigens. Seroprevalence and confidence intervals (95% CI) presented as percentages.

Family	Genus	Antigen	N ^1^	Pos.^2^	Seroprevalence %
*Paramyxoviridae*	*Henipavirus*	HeV	645	33	5.1 (3.6–7.1)
		CedV	637	34	5.3 (3.8–7.3)
		NiV	648	9	1.3 (0.7–2.6)
	*Rubulavirus*	TioV	653	17	2.6 (1.6–4.1)
*Coronaviridae*	*Betacoronavirus*	SARS-CoV	645	38	5.8 (4.3–7.9)
		MERS-CoV	659	10	1.5 (0.8–2.7)

^1^ Total number of samples that were successfully tested. ^2^ Positives: number of samples above the calculated MFI threshold (1000 MFI).

**Table 4:** Seroprevalence per antigen per species. Tested samples (N), followed by the number of positives () are shown. Seroprevalence (SeP) and 95% confident intervals (CI) are presented as percentages. Last two columns present the seroprevalence range for each species. Estimates were not calculated for species with positive results but total sample sizes under 20 individuals (NC). Antigen abbreviations are as follows: Hendra virus (HeV), Nipah virus (NiV), Cedar virus (CedV), Tioman virus (TioV), SARS coronavirus (SARS-CoV), and MERS coronavirus (MERS-CoV).

	Paramyxovirus Antigens								Betacoronavirus Antigens				Overall Summary	
	HeV		CedV		NiV		TioV		SARS-CoV		MERS-CoV			
Species	N	SeP	N	SeP	N	SeP	N	SeP	N	SeP	N	SeP	PaV Range	CoV Range
*C. gouldii*	66(2)	3 (0.8–10.4)	66(4)	6 (2.4–14.6)	68(2)	3 (0.8–10)	68(4)	6 (2.3–14.2)	66(2)	3 (0.8–10.4)	68(2)	3 (0.8–10.1)	3–6	3
*C. morio*	60(5)	8.3 (3.6–18.0)	58(6)	10.3 (4.8–20.7)	59(0)		58(2)	3.3 (0.9–11.5)	59(4)	6.7 (2.6–16)	60(1)	1.6 (0.2–8.7)	3.3–10.3	1.6–6.7
*F. mackenziei*	7(7)	NC	7(7)	NC	7(3)	NC	7(0)		7(0)		7(0)		NC	
*N. geoffroyi*	27(0)		26(0)		26(1)	3.8 (0.6–1.8)	26(0)		26(0)		27(0)		NC	
*N. gouldi*	69(2)	3 (0.8–10)	63(2)	1.6 (0.3–8.4)	67(0)		69(0)		67(7)	10 (5.1–20)	70(0)		1.6–3	10
*N. major*	5(0)		5(0)		5(0)		5(1)	NC	5(2)	NC	4(0)		NC	NC
*S. balstoni*	2(0)		2(0)		2(0)		2(0)		2(0)		2(0)			
*V. baverstocki*	1(0)		1(0)		1(0)		1(0)		1(0)		1(0)			
*V. regulus*	147(7)	4.8 (2.3–9.5)	146(9)	6.1 (3.2–11.3)	150(1)	0.6 (0.1–3.7)	154(2)	1.3 (0.3–4.6)	150(9)	6 (3–11)	155(4)	2.5 (1.0–6.4)	0.6–6.1	2.5–6
*A. australis*	11(0)		11(0)		11(0)		11(1)	NC	11(1)	NC	11(0)		NC	NC
*O. sp*	1(0)		1(0)		1(0)		1(0)		1(0)		1(0)			

**Table 5:** Viral shedding prevalence of three viral families in 11 species of microbats of the South West Botanical Province of Western Australia. Total number of samples tested (N), prevalence values (%), confidence interval (95% CI) and number of positive samples () are shown.

Family	Species	N	*Adenoviridae*	*Coronaviridae*	*Paramyxoviridae*
Vespertilionidae	*C. gouldii*	232	17 (13–23) (41)	25 (20–31) (59)	4 (2–7) (10)
	*C. morio*	45	16 (8–29) (7)	36 (24–51) (17)	9 (3–21) (4)
	*F. mackenziei*	11		NC^1^ (2)	
	*N. geoffroyi*	51	4 (1–13) (2)	4 (1–13) (2)	2 (0.3–10) (1)
	*N. gouldi*	56	1.8 (0.3–9) (1)	3.5 (1–12) (2)	
	*N. major*	10			
	*S. balstoni*	9			NC^1^ (1)
	*V. baverstocki*	4		NC^1^ (2)	
	*V. regulus*	141	8 (4–13) (11)	12 (8–18) (17)	1.4 (0.4–5) (2)
Molossidae	*A. australis*	9			
	*O. sp*	3		NC^1^ (1)	

^1^ Not calculated—calculations were not made for species with total sample sizes below 20 individuals.

## Supplementary Materials

The following are available online at https://www.mdpi.com/1999-4915/11/12/1157/s1, Table S1: GenBank accession numbers for representative sequences produced by this study, Table S2: GenBank accession numbers for outgroup sequences used in the construction of the adenovirus, coronavirus and paramyxovirus phylogenetic trees, Figure S1: Maximum likelihood phylogenetic analysis of the spike protein amino acid sequences derived from five coronavirus genomes.
