# Supplementary material for: Viral Diversity of Microbats within the South West Botanical Province of Western Australia
Source: Viruses. 2019 Dec 13;11(12):1157. doi: 10.3390/v11121157 (PMC6950384; doi:10.3390/v11121157)
Supplement: Supplementary file 1 [file viruses-11-01157-s001.zip › Figure S1_journal_Reviewed.docx]

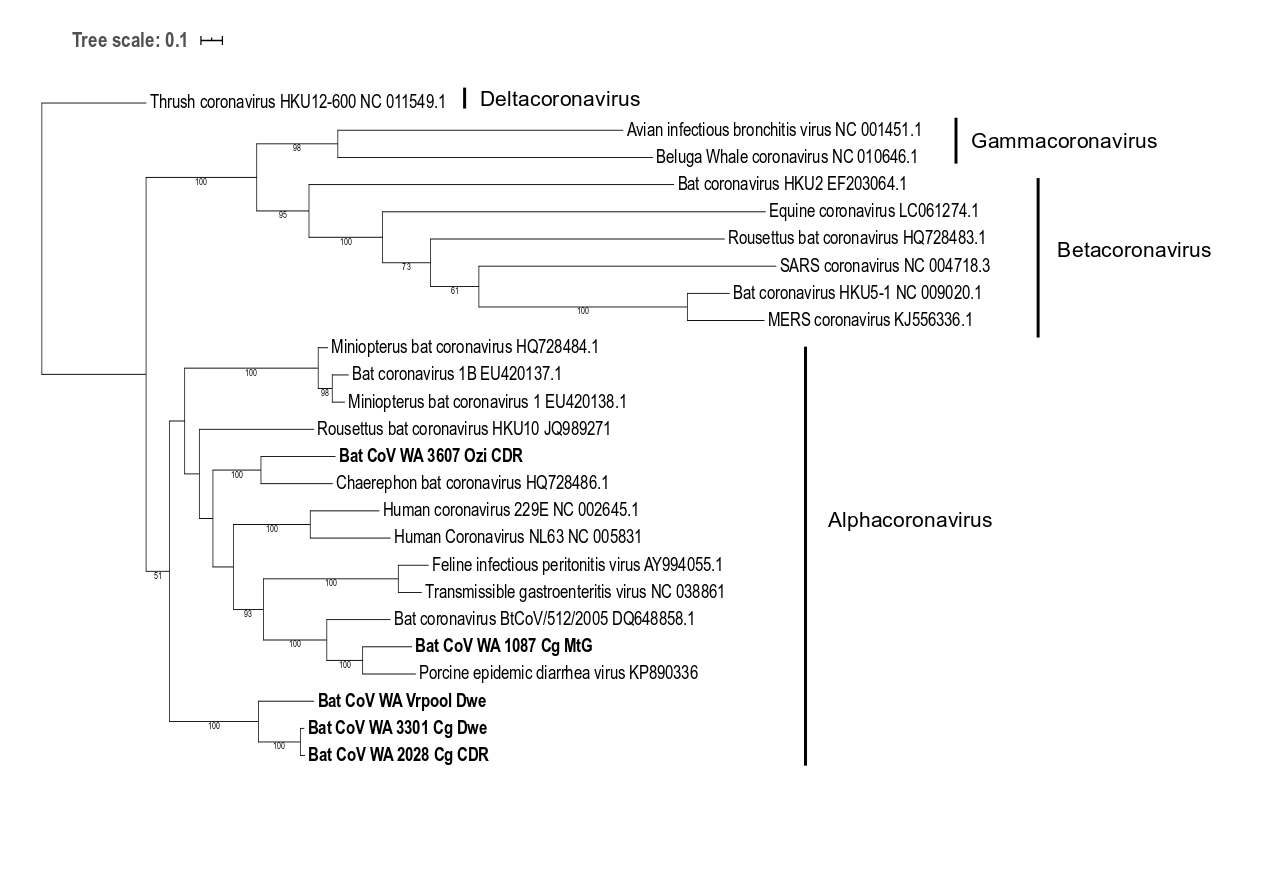


Figure S1. Maximum likelihood phylogenetic analysis of the spike protein amino acid sequences derived from five coronavirus genomes. The tree was constructed in RAxML using the PROTGAMMAWAG model with 1,000 bootstraps; supports above 50% are shown below each branch. Naming convention for the sequences generated in this study represent the unique ID for each individual followed by the first letter of the genus, the first letter of the species and the geographical trapping site.
