# Supplementary material for: Viral Diversity of Microbats within the South West Botanical Province of Western Australia
Source: Viruses. 2019 Dec 13;11(12):1157. doi: 10.3390/v11121157 (PMC6950384; doi:10.3390/v11121157)
Supplement: Supplementary file 1 [file viruses-11-01157-s001.zip › Table S1.docx]

**Table S1**: GenBank accession numbers for representative sequences produced by this study.

| **Accession number** | **Viral family** | **Strain name** | **Host species** | **Names used for sequence analyses** | **Phylogenetic clade it represents** |
| --- | --- | --- | --- | --- | --- |
| MN583338 | *Adenoviridae* | BatAdV_WA01 | *Chalinolobus morio* | Bat_AdV_WA_1066_Cm_CDR | WA AdV I |
| MN583339 | *Adenoviridae* | BatAdV_WA02 | *Chalinolobus morio* | Bat_AdV_WA_3464_Cm_Fra | WA AdV I |
| MN583340 | *Adenoviridae* | BatAdV_WA03 | *Chalinolobus gouldii* | Bat_AdV_WA_1068_Cm_MtG | WA AdV II |
| MN583341 | *Adenoviridae* | BatAdV_WA04 | *Nyctophilus gouldi* | Bat_AdV_WA_3446_Ng_Don | WA AdV II |
| MN583342 | *Adenoviridae* | BatAdV_WA05 | *Chalinolobus gouldii* | Bat_AdV_WA_2055_Cg_CDR | WA AdV III |
| MN583343 | *Adenoviridae* | BatAdV_WA06 | *Chalinolobus gouldii* | Bat_AdV_WA_3302_Cg_Dwe | WA AdV III |
| MN583344 | *Adenoviridae* | BatAdV_WA07 | *Chalinolobus gouldii* | Bat_AdV_WA_3301_Cg_Dwe | WA AdV VI |
| MN583345 | *Adenoviridae* | BatAdV_WA08 | *Chalinolobus gouldii* | Bat_AdV_WA_3206_Cg_Dry | NA |
| MN583346 | *Adenoviridae* | BatAdV_WA09 | *Vespadelus regulus* | Bat_AdV_WA_3270_Vr_Dwe | WA AdV V |
| MN583347 | *Adenoviridae* | BatAdV_WA10 | *Vespadelus regulus* | Bat_AdV_WA_3153_Vr_Dry | WA AdV V |
| MN583348 | *Adenoviridae* | BatAdV_WA11 | *Vespadelus regulus* | Bat_AdV_WA_3323_Vr_Dwe | WA AdV V |
| MN583333 | *Adenoviridae* | BatAdV_WA12 | *Nyctophilus geoffroyi* | Bat_AdV_WA_3091_Ngf_Two | NA |
| MN602054 | *Coronaviridae* | BatCoV_WA01 | *Chalinolobus gouldii* | Bat_CoV_WA_1014_Cg_CDR | WA CoV I |
| MN602055 | *Coronaviridae* | BatCoV_WA02 | *Chalinolobus gouldii* | Bat_CoV_WA_2092_Cg_MtG | WA CoV I |
| MN602056 | *Coronaviridae* | BatCoV_WA03 | *Chalinolobus gouldii* | Bat_CoV_WA_2102_Cg_MtG | WA CoV I |
| MN602057 | *Coronaviridae* | BatCoV_WA04 | *Vespadelus regulus* | Bat_CoV_WA_3254_Vr_Dwe | WA CoV I |
| MN602058 | *Coronaviridae* | BatCoV_WA05 | *Chalinolobus gouldii* | Bat_CoV_WA_3296_Cg_Dwe | WA CoV I |
| MN602059 | *Coronaviridae* | BatCoV_WA06 | *Chalinolobus morio* | Bat_CoV_WA_1120_Cm_MtG | WA CoV II |
| MN602060 | *Coronaviridae* | BatCoV_WA07 | *Chalinolobus morio* | Bat_CoV_WA_3456_Cm_Don | WA CoV II |
| MN602061 | *Coronaviridae* | BatCoV_WA08 | *Vespadelus baverstocki* | Bat_CoV_WA_1006_Vb_CDR | WA CoV III |
| MN602062 | *Coronaviridae* | BatCoV_WA09 | *Vespadelus regulus* | Bat_CoV_WA_3042_Vr_Dry | WA CoV III |
| MN602063 | *Coronaviridae* | BatCoV_WA10 | *Vespadelus regulus* | Bat_CoV_WA_3057_Vr_Twi | WA CoV III |
| MN602064 | *Coronaviridae* | BatCoV_WA11 | *Vespadelus regulus* | Bat_CoV_WA_3276_Vr_Dwe | WA CoV III |
| MN602065 | *Coronaviridae* | BatCoV_WA12 | *Vespadelus regulus* | Bat_CoV_WA_3388_Vr_Bla | WA CoV III |
| MN602066 | *Coronaviridae* | BatCoV_WA13 | *Falsistrellus mackenziei* | Bat_CoV_WA_3396_Fm_Bla | WA CoV III |
| MN602067 | *Coronaviridae* | BatCoV_WA14 | *Chalinolobus gouldii* | Bat_CoV_WA_2129_Cg_MtG | WA CoV IV |
| MN602068 | *Coronaviridae* | BatCoV_WA15 | *Ozimops sp* | Bat_CoV_WA_3607_Oz_CDR | NA |
| MN602069 | *Paramyxoviridae* | BatPaV_WA01 | *Vespadelus regulus* | Bat_PaV_WA_3197_Vr_Dry | NA |
| MN602070 | *Paramyxoviridae* | BatPaV_WA02 | *Chalinolobus gouldii* | Bat_PaV_WA_3606_Cg_CDR | NA |
| MN602071 | *Paramyxoviridae* | BatPaV_WA03 | *Scoterepens balstoni* | Bat_PaV_WA_1034_Sb_CDR | NA |
| MN602072 | *Paramyxoviridae* | BatPaV_WA04 | *Vespadelus regulus* | Bat_PaV_WA_3481_Vr_Fran | NA |
| MN602073 | *Paramyxoviridae* | BatPaV_WA05 | *Chalinolobus gouldii* | Bat_PaV_WA_3204_Cg_Dry | WA PaV I |
| MN602074 | *Paramyxoviridae* | BatPaV_WA06 | *Chalinolobus morio* | Bat_PaV_WA_1120_Cm_MtG | WA PaV II |
| MN602075 | *Paramyxoviridae* | BatPaV_WA07 | *Chalinolobus morio* | Bat_PaV_WA_3070_Cm_Twin | WA PaV II |
