# Supplementary material for: Viral Diversity of Microbats within the South West Botanical Province of Western Australia
Source: Viruses. 2019 Dec 13;11(12):1157. doi: 10.3390/v11121157 (PMC6950384; doi:10.3390/v11121157)
Supplement: Supplementary file 1 [file viruses-11-01157-s001.zip › Table S2.docx]

Table S2: GenBank accession numbers for outgroup sequences used to construct phylogenetic trees of *Adenoviridae*, C*oronaviridae* and *Paramyxoviridae.*

| **Sequence name** | **Accession number** | **Viral family** |
| --- | --- | --- |
| Bovine AdV D | NC002685 | *Adenoviridae* |
| Snake AdV 1 | NC009989 | *Adenoviridae* |
| Fowl AdV A | NC001720 | *Adenoviridae* |
| Fowl AdV D | NC000899 | *Adenoviridae* |
| Frog Adv 1 | NC002501 | *Adenoviridae* |
| Turkey AdV 3 | NC001958 | *Adenoviridae* |
| Avian infectious bronchitis virus | NC001451 | C*oronaviridae* |
| Beluga whale CoV | NC010646 | C*oronaviridae* |
| Munia CoV | NC011550 | C*oronaviridae* |
| Thrush CoV | NC011549 | C*oronaviridae* |
| Human BetaCoV 2c | JX869059 | C*oronaviridae* |
| Bat CoV HKU5-1 | NC009020 | C*oronaviridae* |
| Bat CoV HKU4-1 | NC009019 | C*oronaviridae* |
| Bat CoV CoV000 | EU834950 | C*oronaviridae* |
| SARS CoV | NC004718 | C*oronaviridae* |
| Bat Cov Gabon | JX174638 | C*oronaviridae* |
| Bat CoV GhanaKwam | FJ710052 | C*oronaviridae* |
| Bat CoV HKU9-1 | NC009021 | C*oronaviridae* |
| Bat CoV KY06 | HQ728483 | C*oronaviridae* |
| Human CoV HKU1 | NC006577 | C*oronaviridae* |
| Mouse hepatitis virus MHV-A59 | NC001846 | C*oronaviridae* |
| Human metapneumovirus | AF371337 | *Paramyxoviridae* |
| Avian metapneumovirus | AY590688 | *Paramyxoviridae* |
| Human respiratory syncytial virus | HRU39662 | *Paramyxoviridae* |
| Bovine respiratory syncytial virus | AF295543 | *Paramyxoviridae* |
| Cedar virus | JQ001776 | *Paramyxoviridae* |
| Hendra virus | AF017149 | *Paramyxoviridae* |
| Nipah virus | AF212302 | *Paramyxoviridae* |
| Sendai virus | AB195968 | *Paramyxoviridae* |
| Human parainfluenza virus 1 | AF457102 | *Paramyxoviridae* |
| Human parainfluenza virus 3 | EU424062 | *Paramyxoviridae* |
| Human parainfluenza virus 4a | AB543336 | *Paramyxoviridae* |
| Human parainfluenza virus 2 | AF533010 | *Paramyxoviridae* |
| Bovine parainfluenza virus 3 | AF178654 | *Paramyxoviridae* |
| Simian Agent 10 | HM583801 | *Paramyxoviridae* |
| Mumps virus | AB040874 | *Paramyxoviridae* |
| Porcine rubulavirus | BK005918 | *Paramyxoviridae* |
| Mapuera virus | EF095490 | *Paramyxoviridae* |
| Simian parainfluenza virus 5 | AF052755 | *Paramyxoviridae* |
| Tioman virus | AF298895 | *Paramyxoviridae* |
| Menangle virus | AF326114 | *Paramyxoviridae* |
| Avian PaV 2 | EU338414 | *Paramyxoviridae* |
| Avian PaV 8 | FJ215863 | *Paramyxoviridae* |
| Avian PaV 6 | EF569970 | *Paramyxoviridae* |
| Newcastle disease virus | AF077761 | *Paramyxoviridae* |
| Avian PaV 9 | EU910942 | *Paramyxoviridae* |
| Avian PaV 3 | EU403085 | *Paramyxoviridae* |
| Avian PaV 4 | EU877976 | *Paramyxoviridae* |
| Avian PaV 7 | FJ231524 | *Paramyxoviridae* |
| Avian PaV 5 | GU206351 | *Paramyxoviridae* |
